# Upregulation of TMEM16A Protein in Bronchial Epithelial Cells by Bacterial Pyocyanin

**DOI:** 10.1371/journal.pone.0131775

**Published:** 2015-06-29

**Authors:** Emanuela Caci, Paolo Scudieri, Emma Di Carlo, Patrizia Morelli, Silvia Bruno, Ida De Fino, Alessandra Bragonzi, Ambra Gianotti, Elvira Sondo, Loretta Ferrera, Alessandro Palleschi, Luigi Santambrogio, Roberto Ravazzolo, Luis J. V. Galietta

**Affiliations:** 1 Istituto Giannina Gaslini, Genova, Italy; 2 Anatomic Pathology and Molecular Medicine, Department of Medicine and Sciences of Aging, "G. d'Annunzio" University, Chieti, Italy; 3 Ce.S.I. Biotech, Aging Research Center, "G. d'Annunzio" University Foundation, Chieti, Italy; 4 Dept. of Experimental Medicine, DIMES, University of Genova, Genova, Italy; 5 Infection and Cystic Fibrosis Unit, IRCCS San Raffaele Scientific Institute, Milano, Italy; 6 Thoracic Surgery and Lung Transplantation Unit, Fondazione IRCCS “Ca’ Granda” Ospedale Maggiore Policlinico, University of Milan, Milano, Italy; 7 DINOGMI, University of Genova, Genova, Italy; University of Alabama at Birmingham, UNITED STATES

## Abstract

Induction of mucus hypersecretion in the airway epithelium by Th2 cytokines is associated with the expression of TMEM16A, a Ca^2+^-activated Cl^-^ channel. We asked whether exposure of airway epithelial cells to bacterial components, a condition that mimics the highly infected environment occurring in cystic fibrosis (CF), also results in a similar response. In cultured human bronchial epithelial cells, treatment with pyocyanin or with a *P*. *aeruginosa* culture supernatant caused a significant increase in TMEM16A function. The Ca^2+^-dependent Cl^-^ secretion, triggered by stimulation with UTP, was particularly enhanced by pyocyanin in cells from CF patients. Increased expression of TMEM16A protein and of MUC5AC mucin by bacterial components was demonstrated by immunofluorescence in CF and non-CF cells. We also investigated TMEM16A expression in human bronchi by immunocytochemistry. We found increased TMEM16A staining in the airways of CF patients. The strongest signal was observed in CF submucosal glands. Our results suggest that TMEM16A expression/function is upregulated in CF lung disease, possibly as a response towards the presence of bacteria in the airways.

## Introduction

The airway epithelium displays a series of defense mechanisms against bacteria and viruses that reach the airway surface with the inhaled air. In particular, the airways are covered by a liquid and a mucus layer [1]. The liquid layer, known as the periciliary liquid (PCL), is maintained at a fixed thickness of nearly 7 microns through the balance between fluid/electrolyte secretion and absorption [1,2]. PCL is important to maintain the mucus layer at the optimal distance from the airway surface in order to be moved by the beating of cilia [2]. Major players in the homeostasis of PCL volume and composition are the Na^+^ and Cl^-^ channels localized on the apical membrane of airway epithelial cells [1,3]. The epithelial sodium channel ENaC is responsible for Na^+^ absorption [4]. A significant fraction of chloride secretion is instead mediated by the cystic fibrosis transmembrane conductance regulator (CFTR), a channel whose activity is elicited by cAMP-dependent phosphorylation [5]. CFTR loss of function is the basic defect in cystic fibrosis (CF). Lack of secretion through CFTR causes depletion of the PCL and impairment of mucociliary transport [2]. As a consequence, the airways are clogged by a highly viscous mucus that becomes the niche for bacterial colonization. Since CFTR pore is permeable to bicarbonate, high viscosity of mucus in CF may also depend on defective bicarbonate secretion. Indeed, bicarbonate is required to facilitate mucus release and expansion [6–8].

In addition to CFTR, the airway epithelium also expresses TMEM16A, a Ca^2+^-activated Cl^-^ channel [9–12]. The precise role of Ca^2+^-dependent Cl^-^ secretion in the airways is not clear. However, TMEM16A expression is strongly upregulated by the Th2 cytokines IL-4 and IL-13 [9,12,13]. Such cytokines also induce mucous metaplasia, i.e. increased abundance of mucus-secreting goblet cells [14–17]. This suggests a possible link between TMEM16A and mucus, a relationship that may be explained with the ability of this channel to transport bicarbonate [18].

The expression and role of TMEM16A in CF are not clear. We previously found no difference in TMEM16A expression and function between CF and non-CF cultured bronchial epithelial cells [12]. Also, both types of cells responded equally to IL-4 with upregulation of TMEM16A expression and Ca^2+^-dependent Cl^-^ secretion. However, CF airway epithelial cells are exposed to bacteria in vivo. Bacterial components may trigger active responses in epithelial cells, with altered expression of cell proteins. In particular, pyocyanin, a major virulence factor of *P*. *aeruginosa*, and bacterial supernatants were found to induce mucus metaplasia and MUC5AC expression [19,20]. Since there seems to be a relationship between TMEM16A expression and mucus hypersecretion [12,13], we asked whether TMEM16A is also affected by bacterial components. We found that pyocyanin, as well as whole bacterial supernatants from *P*. *aeruginosa*, increase Ca^2+^-dependent Cl^-^ secretion along with expression of TMEM16A and of MUC5AC mucin.

## Materials and Methods

### Cell culture

The isolation, culture, and differentiation methods of primary bronchial epithelial cells were previously described in detail [12]. Briefly, epithelial cells were obtained from mainstem human bronchi, derived from CF and non-CF individuals undergoing lung transplant. For this study, cells were obtained from two CF patients (homozygous for F508del mutation) and two non-CF patients. Cells were detached by overnight incubation of bronchi at 4°C in a solution containing protease XIV. Epithelial cells were then cultured in a serum-free medium (LHC9 mixed with RPMI 1640, 1:1) supplemented with various hormones and supplements. This medium favors cell number amplification. When cells were derived from CF patients, the culture medium contained in the first days a complex mixture of antibiotics (usually colistin, piperacillin, and tazobactam) to eradicate bacteria. The collection of bronchial epithelial cells and their study to investigate the mechanisms of transepithelial ion transport were specifically approved by the Ethics Committee of the Istituto Giannina Gaslini following the guidelines of the Italian Ministry of Health. Each patient provided informed consent to the study using a form that was also approved by the Ethics Committee.

To obtain differentiated epithelia, cells were seeded at high density on porous membranes (12 mm Snapwell inserts, Corning, code 3801). After 24 hours, the serum-free medium was replaced with DMEM/Ham’s F12 containing 2% fetal bovine serum plus hormones and supplements. Differentiation of cells into a tight epithelium was checked by measuring transepithelial electrical resistance and potential difference with an epithelial voltohmmeter (EVOM1, World Precision Instruments). The medium was replaced daily on both sides of permeable supports up to 8–10 days (liquid-liquid culture, LLC). Subsequently the apical medium was totally removed and the cells received nutrients only from the basolateral side (air-liquid culture, ALC). This condition favored a further differentiation of the epithelium. Cells were maintained under ALC for 2–3 weeks before experiments.

To test bacterial components, we added 100 μl of PBS to the apical side of the epithelium with and without bacterial components. Pyocyanin concentration was 60 μM whereas the bacterial supernatant was diluted 1:8 into PBS. Higher concentrations of pyocyanin and supernatant were not studied because they caused a marked decrease in epithelial resistance. To prepare the bacterial supernatant, the CF clinical *P*. *aeruginosa* strain used in this study was grown in M9 medium at 37°C to late log phase and a cell-free culture supernatant was obtained, as described previously [21]. Briefly, the broth culture was centrifuged at 10,000 rpm for 50 min. The supernatant containing bacterial exoproducts was sterilized by passage through a 0.22-micron polymer filter (Corning) and kept at -80°C until used.

### Short-circuit current recordings

Snapwell inserts carrying differentiated bronchial epithelia were mounted in a vertical diffusion chamber resembling a Ussing chamber with internal fluid circulation. Both apical and basolateral hemichambers were filled with 5 ml of a solution containing (in mM): 126 NaCl, 0.38 KH_2_PO_4_, 2.13 K_2_HPO_4_, 1 MgSO_4_, 1 CaCl_2_, 24 NaHCO_3_, and 10 glucose. Both sides were continuously bubbled with a gas mixture containing 5% CO_2_ - 95% air and the temperature of the solution was kept at 37°C. The transepithelial voltage was short-circuited with a voltage-clamp (DVC-1000, World Precision Instruments) connected to the apical and basolateral chambers via Ag/AgCl electrodes and agar bridges (1 M KCl in 1% agar). The offset between voltage electrodes and the fluid resistance were canceled before experiments. The short-circuit current was recorded with a PowerLab 4/25 (ADInstruments) analogical to digital converter connected to a Macintosh computer.

### Intracellular Ca^2+^ determination

Bronchial epithelial cells on Snapwell inserts were washed in PBS and then loaded for 1 h with 5 μM Fluo-4/AM (Life Technologies) in PBS. Loading solution, applied to both sides of the epithelium, also contained 10 mM glucose, 0.5 mM sulfinpyrazone, and 1% fetal bovine serum. After loading, cells were rapidly washed and equilibrated in PBS containing 10 mM glucose and 0.5 mM sulfinpyrazone (250 μl apical, 2 ml basolateral). A six-well plate carrying the Snapwell inserts with fluorescent cells was then introduced in a BMG FLUOstar Galaxy microplate reader. The assay consisted of continuous reading of cell fluorescence (excitation: 485 nm; emission: 520 nm) for 40 s (sampling time: 0.2 s). At 4 s, the reader injected on the apical side 250 μl of PBS containing 10 mM glucose, 0.5 mM sulfinpyrazone, and 200 μM UTP. Each trace was corrected for the background (Snapwell inserts without cells).

### Western blot assays

Cells were lysed in RIPA buffer 1X (50 mM Tris-HCl pH 7.4, 150 mM NaCl, 1% Triton X-100, 0.5% sodium deoxycholate, 0.1% SDS) containing Complete Protease Inhibitor Cocktail (Roche, NJ, USA). The protein concentration in lysates was quantified using the Quantum Protein Assay kit (Euroclone). Total lysates were electrophoresed (30 μg per lane) with NuPAGE Novex Bis-Tris 4–12% gel (Life Technologies) and transferred to a nitrocellulose membrane (Bio-Rad, Hercules, CA, USA) for Western blotting. The TMEM16A protein was immunodetected with the r [SP31] rabbit monoclonal antibody (ab64085, Abcam; 1:1000). The secondary antibody was anti-rabbit HRP (Millipore; 1:5000). Membranes were also stripped with the Restore Western Blot Stripping Buffer (Thermo Fisher Scientific Inc.) and incubated with the mouse monoclonal C464.8 (Merck Millipore) antibody against Na^+^/K^+^-ATPase β1 (1:6000) followed by anti-mouse HRP-conjugated secondary antibody (Ab 97023, Abcam; 1:10000). All antibodies were dissolved in 5% skimmed-milk in Tris-buffered saline—Tween 20. Protein bands were visualized using the ECL Advanced Western Blotting Detection Kit (GE Healthcare Europe, Little Chalfont, UK). Direct recording of the chemiluminescence was performed using the Molecular Imager ChemiDoc XRS System (Bio-Rad).

### Immunofluorescence

Primary human bronchial epithelial cells on Snapwell permeable supports were rinsed twice with PBS and fixed by adding 200 μl of 10% neutral buffered formalin (05-01005Q, Bio-Optica) for 10 minutes. After three washings with PBS, cells were subjected to antigen retrieval with 10 mM citrate buffer pH = 6 heated to 95°C in a microwave for 5 minutes. Samples were then cooled to room temperature in PBS and permeabilized with PBS-Triton X-100 0.3% for 5 minutes. After washing, cells were blocked with 1% bovine serum albumin (BSA) in PBS for 2 hours and then incubated overnight at 4°C with 200 μl of primary antibodies diluted in PBS containing BSA 1% and Triton X-100 0.3%. The following antibodies and dilutions were used: rabbit monoclonal anti-TMEM16A [SP31] (ab64085, Abcam) at 1:200, mouse IgG2B anti-acetylated tubulin (7451, Sigma Aldrich) at 1:300, NCL-HGM-45M1 mouse IgG1 anti-MUC5AC (Novocastra) at 1:50.

Following incubation with primary antibodies, cells were rinsed three times in PBS and incubated with 200 μl of a solution of secondary goat anti-rabbit Alexa Fluor 488, goat anti-mouse IgG1 Alexa Fluor 546 and goat anti-mouse IgG2B Alexa Fluor 633 antibodies (Life Technologies) diluted at 1:200 in PBS-BSA 1% for 1 hour in the dark. After further 3 washes in PBS, the porous membrane carrying the cells was cut from the plastic support of the Snapwell, placed on microscope slides and mounted with Fluoroshield with 4',6-diamidino-2-phenylindole (DAPI) (Sigma-Aldrich) to stain cell nuclei.

Confocal microscopy was performed using a laser scanning spectral confocal microscope TCS SP2-AOBS (Leica Microsystems, Heidelberg, Germany). Image analysis was performed using Leica and ImageJ software. TMEM16A positive-cells, goblet cells, and total number of cells (nuclei) were manually counted in *xy* fields having the size of 375 x 375 μm (at least 4 fields per condition). Each field contained 904 ± 23 cells (at least 4,000–5,000 cells were counted per condition).

### Histology and immunohistochemistry

Tissue samples were obtained from four CF patients (with the following mutations: F508del/F508del, F508del/R1066H, F508del/G542X, F508del/1717-1G>A) and from two non-CF patients. Samples were fixed in 4% formalin and embedded in paraffin. For histology, paraffin-embedded samples were sectioned at 3 μm and stained with hematoxylin and eosin (H&E). For immunohistochemistry on the formalin-fixed, paraffin-embedded samples, sections were deparaffinized, subjected to antigen retrieval, treated with H_2_O_2_/3% for 5 minutes, to inhibit endogenous peroxidase, and incubated for 30 minutes with TMEM16A (rabbit anti-human TMEM16A, clone SP31 Abcam, Cambridge, UK) antibody. Immune complexes were detected using the Bond Polymer Refine Detection Kit according to the manufacturer’s protocol (Leica Biosystems, Wetzlar, Germany).

### Statistics

Data are presented as representative traces or as mean values ± SEM. Statistical analysis was done with one-way ANOVA using the InStat (GraphPad) software.

## Results

Human bronchial epithelial cells were allowed to differentiate as a polarized epithelium on porous supports and then incubated with pyocyanin 60 μM for 24 and 72 hours or with bacterial supernatant for 24 hours. After treatment, cells were studied in short-circuit current recordings. The experimental protocol included block of ENaC with amiloride (10 μM), stimulation of CFTR activity with CPT-cAMP (100 μM), block of CFTR with CFTR_inh_-172 (10 μM), and stimulation of Ca^2+^-dependent Cl^-^ secretion with UTP (100 μM). All compounds were added in the apical chamber. CPT-cAMP was also added in the basolateral chamber.

**Fig 1:**
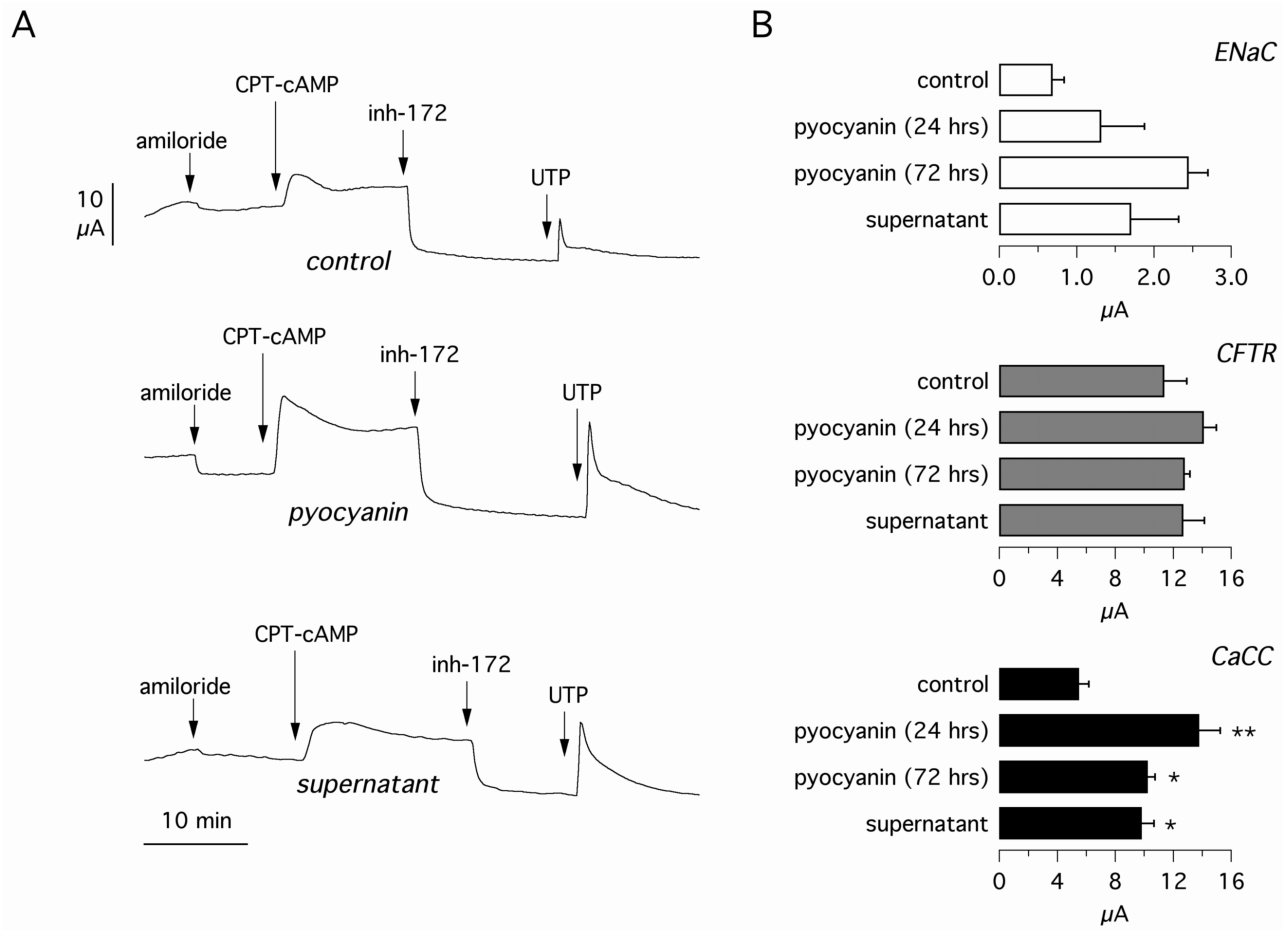
Effect of bacterial components on ion transport in cultured bronchial epithelial cells. **(A)** Representative short-circuit recordings on cultured non-CF human bronchial epithelial cells. Cells were treated for 24 hours with and without pyocyanin (60 μM) or bacterial culture supernatant. Arrows indicate the time of addition of amiloride (10 μM), CPT-cAMP (100 μM), CFTR_inh_-172 (10 μM), and UTP (100 μM). **(B)** Bar graphs report the amplitude of currents measured under the indicated conditions. ENaC graph: amiloride-sensitive current; CFTR graph: amplitude of current drop produced by addition of CFTR_inh_-172; CaCC graph: maximal current elicited by addition of UTP, which triggers the activation of Ca^2+^-activated Cl^-^ channels. Each bar is the mean plus SEM of 7–8 experiments. *, p < 0.05; **, p < 0.01 vs. control.

Fig 1 shows data (representative traces and bar graphs) from experiments on non-CF epithelia. Incubation with pyocyanin and supernatant elicited an increase in Ca^2+^-dependent Cl^-^ secretion. The largest effect, a more than 2-fold increase in the current peak elicited by UTP, was observed with pyocyanin at 24 hours. We also observed an increase in ENaC activity (amiloride-sensitive current) with pyocyanin and bacterial supernatant but these effects did not reach statistical significance (Fig 1). CFTR function was instead unaffected by treatments.

**Fig 2:**
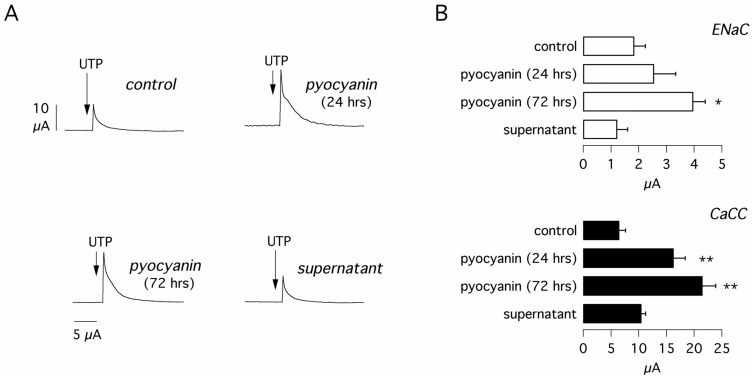
Upregulation of Ca^2+^-dependent Cl^-^ secretion by pyocyanin in CF cells. **(A)** Representative short-circuit recordings on cultured CF bronchial epithelial cells. Cells were treated with pyocyanin (60 μM, 24 and 72 hours) or with bacterial supernatant (24 hours). The experiments were done as shown in Fig 1 (sequential addition of amiloride, CPT-cAMP, CFTR_inh_-172, and UTP). However, for simplicity, only the response to UTP is shown. **(B)** Bar graphs show the values measured for ENaC and CaCC. CFTR currents were very small and not affected by treatments. Each bar is the mean plus SEM of 8–9 experiments. *, p < 0.05; **, p < 0.01 vs. control.

Pyocyanin was also effective in CF bronchial epithelial cells, particularly at 72 hours (Fig 2). Under this condition, the UTP peak was increased three-fold. Interestingly, the UTP-dependent current measured in CF cells at 72 hours was significantly larger than that measured in non-CF cells (21.5 ± 2.37 vs. 10.25 ± 0.48 μA/cm^2^, p < 0.01). In contrast, the bacterial supernatant was ineffective. Pyocyanin at 72 hours also significantly increased the activity of ENaC in CF cells (Fig 2B). Regarding CFTR, the currents were obviously very small in CF cells under resting conditions (0.86 ± 0.13 μA/cm^2^, not shown) and no change was detected in cells treated with bacterial components. In the same experiments, we also tested IL-4 for comparison. This resulted, as previously reported [12] in a more than eight-fold increase in UTP-dependent current (not shown).

**Fig 3:**
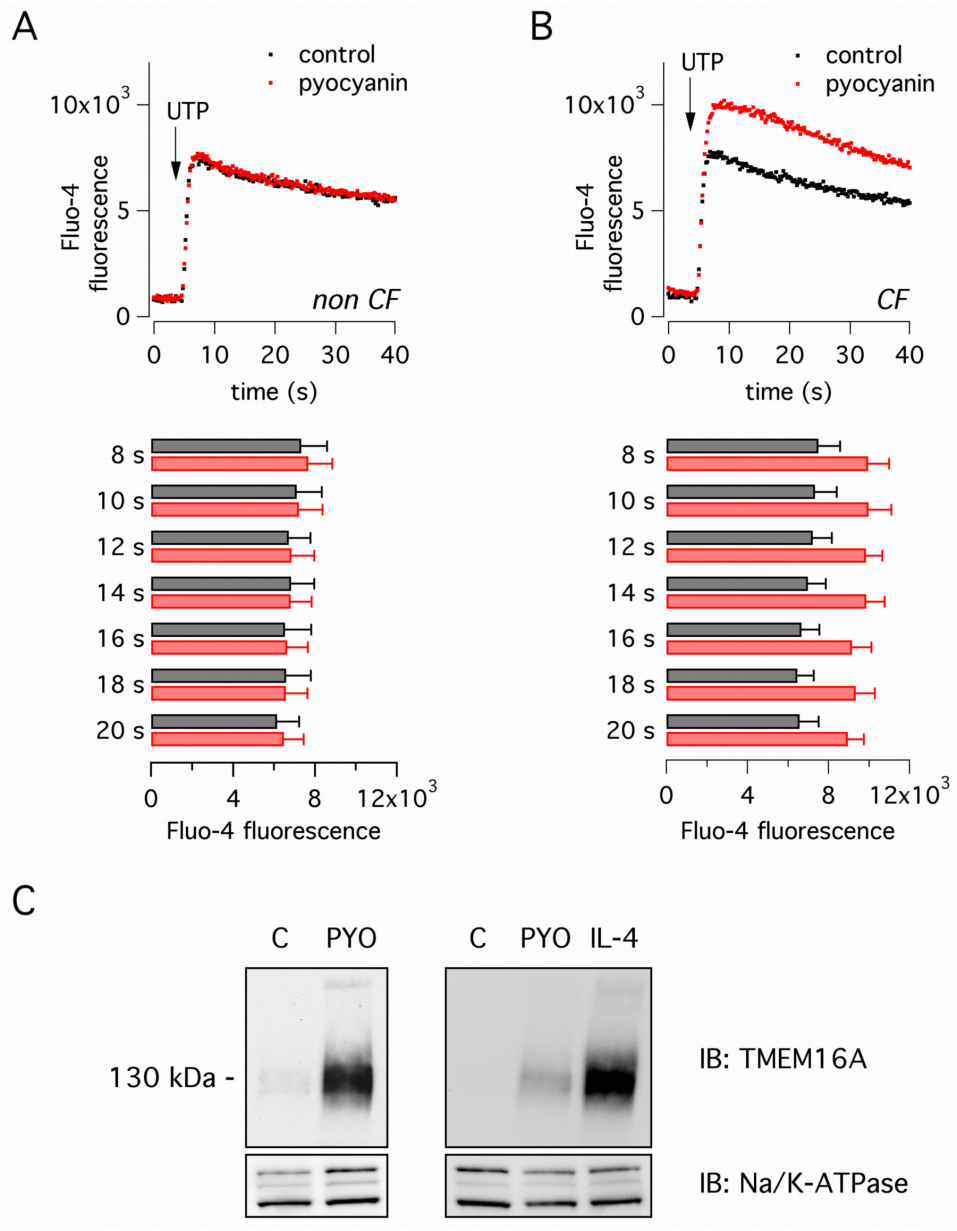
Analysis of pyocyanin mechanism of action. **(A,B)** Cytosolic Ca^2+^ revealed with the fluorescent Fluo-4 probe in non-CF and CF bronchial epithelial cells, with and without treatment with pyocyanin (60 μM, 24 hours). Top graphs show the time-course of Fluo-4 fluorescence before and after acute stimulation with apical UTP (100 μM). Each dot is the mean of three experiments. Bottom graphs show the values of fluorescence measured at the indicated time points. Although the response to UTP in pyocyanin-treated CF cells was larger than in untreated cells, the difference was not statistically significant. **(C)** Western blot analysis of TMEM16A protein expression. Non-CF cells were treated with pyocyanin (60 μM) or IL-4 (10 ng/ml) for 24 hours. Pyocyanin and, to a larger extent, IL-4 increased TMEM16A expression. Each panel is representative of three similar experiments.

The increase in Ca^2+^-dependent Cl^-^ secretion induced by bacterial components could be the result of altered intracellular Ca^2+^ mobilization and/or upregulation of TMEM16A expression. To evaluate the first type of mechanism, we measured intracellular Ca^2+^ with the fluorescent Fluo-4 probe. Stimulation with apical UTP (100 μM) triggered a fast increase in cytosolic Ca^2+^ concentration (Fig 3A and 3B). In non-CF cells, we found that pyocyanin treatment did not alter the amplitude of the UTP-dependent Ca^2+^ increase (Fig 3A). In CF cells treated with pyocyanin, the response to UTP appeared larger with respect to untreated cells but the difference was not statistically significant (Fig 3B). To assess a possible effect of pyocyanin on TMEM16A protein expression, we carried out western blot experiments. In untreated cells, TMEM16A protein expression was weakly detectable. Pyocyanin treatment markedly increased the TMEM16A signal (Fig 3C, left panel). We also compared TMEM16A expression induced by pyocyanin and by IL-4 (Fig 3C, right panel). IL-4 was more effective, in agreement with functional data.

**Fig 4:**
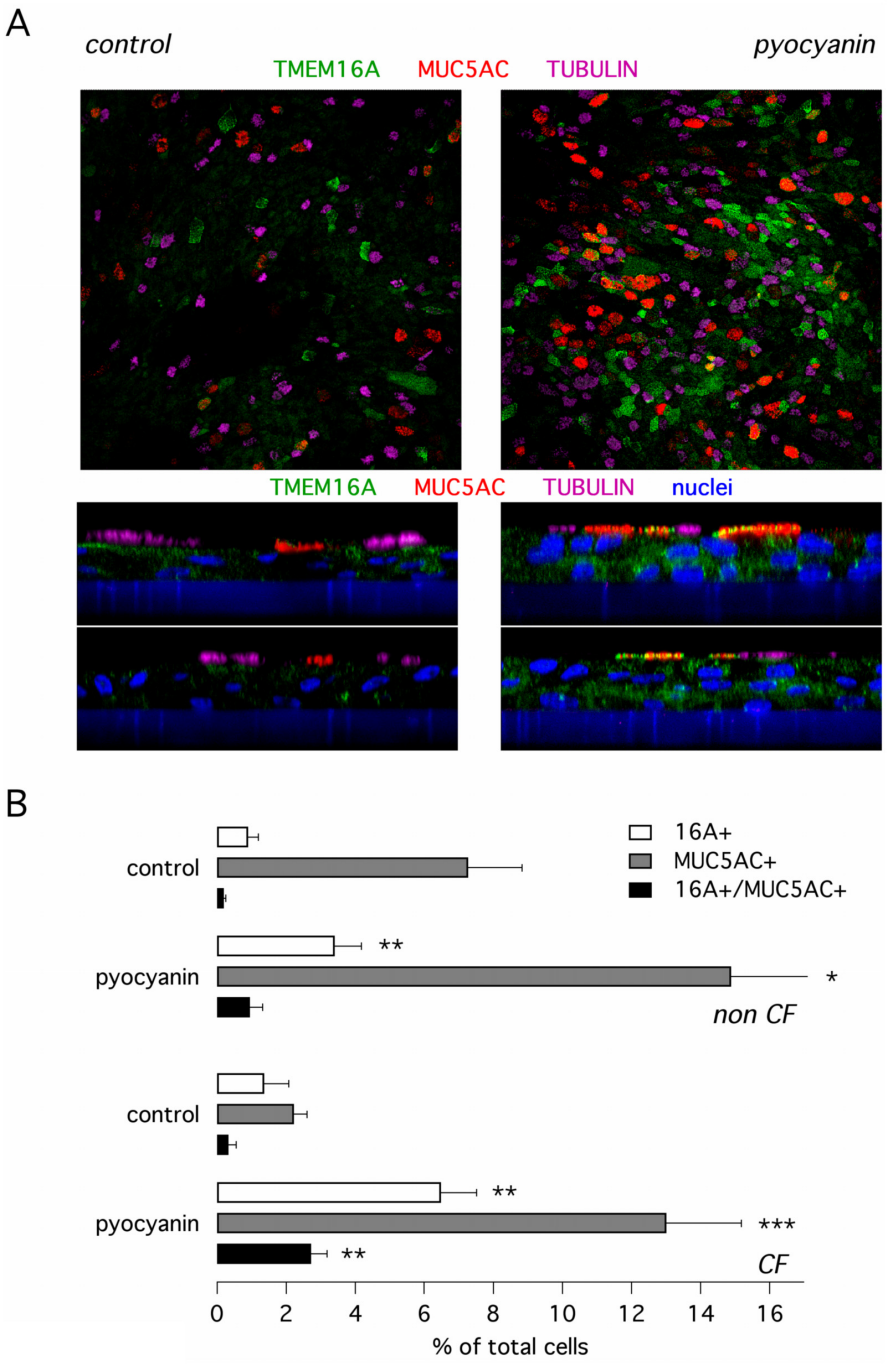
Expression of TMEM16A protein in cultured bronchial epithelial cells. **(A)** Representative images showing detection of TMEM16A (green), MUC5AC (red), and acetylated tubulin (magenta) by immunofluorescence. Images, taken with a confocal microscope, are *xy* (top) and *xz* (bottom) scans of bronchial epithelial cells treated with and without pyocyanin. The *xz* images also report staining of nuclei with DAPI (blue). The blue layer under the cells is the porous membrane of the Snapwell insert. **(B)** Bar graphs report the percentage of cells positive for TMEM16A, for MUC5AC, and for both proteins together. *, p < 0.05; **, p < 0.01 vs. control.

To confirm the upregulation of TMEM16A protein by pyocyanin we used immunofluorescence experiments. Cells were stained with antibodies against TMEM16A, MUC5AC, and acetylated tubulin (for ciliated cells). In agreement with functional data, we found that incubation with pyocyanin increased the percentage of CF and non-CF cells expressing TMEM16A protein (Fig 4). Pyocyanin also increased the percentage of cells expressing MUC5AC mucin. However, only a fraction of MUC5AC-positive cells (nearly 20% in CF epithelia) coexpressed TMEM16A. No co-expression was found for TMEM16A and tubulin. We also found no difference when comparing CF and non-CF cells: both types of cells responded similarly to pyocyanin (Fig 4).

**Fig 5:**
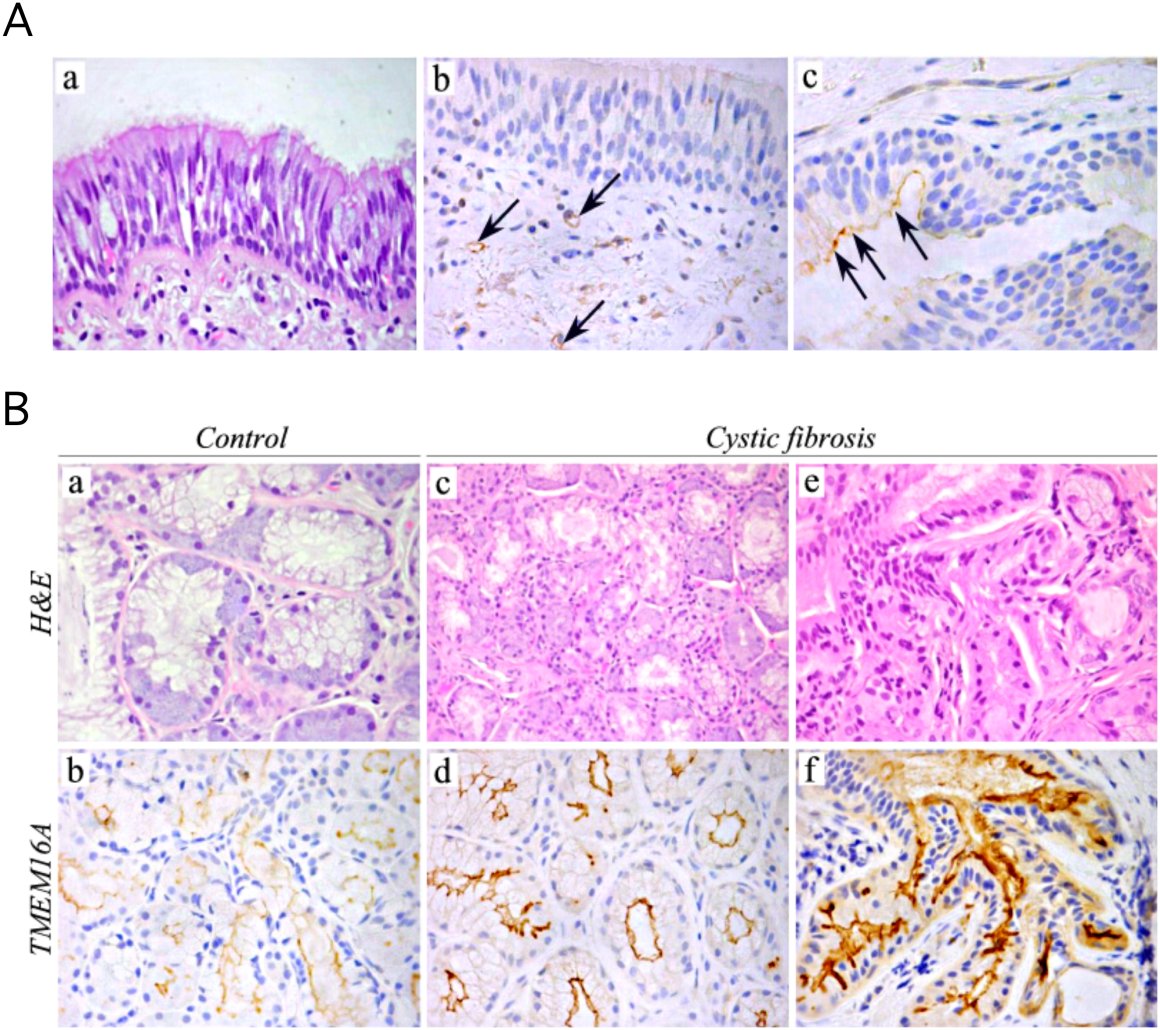
Immunohistochemical detection of TMEM16A in human airways. **(A)** Expression of TMEM16A in the surface epithelium. TMEM16A staining was mostly absent (b), and sometimes scanty (c) in the respiratory epithelium lining bronchi or bronchioles from CF patients (c, arrows). A weak expression was also detectable in microvessels of peri-bronchial connective tissue (b, arrows). Magnification: X400. **(B)** TMEM16A expression was modest in non-CF submucosal glands of non-CF samples (b) but markedly increased in tissues from CF patients (d), with a particularly strong signal (f) in histologically altered glands (e). Magnification: X630 in a, X400 in b-f. Images 4Aa and 4Ba,c,e show hematoxylin and eosin staining.

The *in vitro* induction of TMEM16A expression by bacterial components led us to ask whether a similar response occurs also *in vivo*. Therefore, we performed immunohistochemical analysis of bronchi from CF and non-CF subjects. We found that, in bronchi from non-CF subjects, the expression of TMEM16A in the surface epithelium was undetectable (not shown), whereas in CF bronchi, a slight and unsteady TMEM16A expression was found in the apical membrane of columnar cells of epithelium (Fig 5Ac, arrows). In CF patients, as in non-CF subjects, we also found staining of microvessels located in the peri-bronchial connective tissue (Fig 5Ab, arrows).

TMEM16A expression was barely detectable in the bronchial submucosal glands of non-CF samples (Fig 5Bb). In contrast, in patients with CF, submucosal glands showed a distinct (Fig 5Ad) to strong (Fig 5Af) staining for TMEM16A.

## Discussion

TMEM16A protein contributes to Cl^-^ and bicarbonate secretion in various organs and tissues. In the airways, TMEM16A represents an alternative route for anion transport with respect to CFTR [9,22]. This is particularly relevant for CF, in which lack of CFTR function causes a severe impairment of innate defense mechanisms including mucociliary clearance and bactericidal mechanisms [1,2,23]. Therefore, the extent of TMEM16A expression in CF airways could potentially modify the lung phenotype and be important in determining the efficacy of therapeutic strategies trying to circumvent the CFTR defect.

In previous studies, we and others found that Ca^2+^-dependent Cl^-^ secretion in cultured bronchial epithelia is strongly regulated by IL-4 and IL-13 [24,25], two cytokines that mediate Th2 immune response and induce mucus metaplasia in the airways [14–17]. An increased anion secretion by allergic airway inflammation was also observed in mice [26]. We subsequently found that upregulation of anion secretion by Th2 cytokines is due to increased expression of the TMEM16A chloride channel protein [9]. This phenomenon also occurred when IL-4 was applied to CF bronchial epithelia *in vitro* [12]. However, it is not clear if this also occurs *in vivo* although there is evidence that the immune response to chronic bacterial infection in CF is of the Th2 type [27,28].

To reproduce, at least in part, the conditions of the CF airway epithelium *in vivo*, we treated cultured bronchial epithelial cells with pyocyanin and bacterial supernatant. Interestingly, both treatments resulted in upregulation of Ca^2+^-dependent Cl^-^ secretion, although pyocyanin appeared more effective than the supernatant. The effect on Cl^-^ secretion was not due to altered intracellular Ca^2+^ mobilization but to increased TMEM16A protein expression, a mechanism of action that is similar, although less potent, than that triggered by IL-4 in bronchial epithelial cells (9).

The effect of short-term treatment of pyocyanin on ion transport in a bronchial cell line was previously investigated [29]. This study found that pyocyanin stimulates CFTR function. In our study, we found that chronic exposure of primary bronchial epithelial cells does not affect CFTR function.

In our experiments, pyocyanin also increased the percentage of cells expressing MUC5AC as found previously by others [19]. The underlying mechanism of action of pyocyanin seems to be shared with that of Th2 cytokines [19]. However, in our experiments with pyocyanin the percentage of cells co-expressing MUC5AC and TMEM16A was relatively low compared with the value previously reported for cells treated with IL-4 [12]. This could indicate that Th2 cytokines and pyocyanin signaling cascades are not identical and that significant differences may be responsible for the different effect on the extent and site of expression of TMEM16A and MUC5AC.

Interestingly, we found that pyocyanin treatment affected ENaC function, with a significant increase in amiloride-sensitive current in CF cells at 72 hours. This effect could result in higher Na^+^ ad fluid absorption. Since pyocyanin also increases Cl^-^ secretion through TMEM16A, it remains to determined which process prevails *in vivo*.

We also investigated TMEM16A expression by immunohistochemical analysis of bronchi from CF and non-CF subjects. Intriguingly, TMEM16A expression was undetectable in the airway surface epithelium of non-CF samples. This contrasts with the presence of a Ca^2+^-dependent Cl^-^ secretion that has been reported also in vivo for human airways. The absence of a TMEM16A signal could be explained with the inability of our antibody to detect a relatively low, but functionally relevant, expression of this protein. In contrast, TMEM16A staining was observed in the surface epithelium of CF bronchi, as a possible result of increased expression triggered by bacterial colonization and inflammation. Actually, the non-homogeneous expression of TMEM16A in the surface epithelium of CF airways could be explained with different degrees of infection/inflammation. An unexpected finding of our study was the strong TMEM16A signal in the submucosal glands of CF patients. In addition to surface epithelium, glands are another important site for anion secretion. In particular, CFTR-dependent Cl^-^ and bicarbonate transport is important to support the secretion of mucins and other macromolecules [8,30,31]. In this respect, it has been recently found in CF pigs that lack of CFTR blocks the release of mucus from submucosal glands [8]. This results in the accumulation of mucus strands on the airway surface [8].

Submucosal glands also show a Ca^2+^-dependent anion secretion, probably mediated by TMEM16A [32,33]. The relative role of the two channels, CFTR and TMEM16A, in gland function is unclear, but the former could be involved in basal secretion, whereas the latter could be instead recruited by neurohumoral stimulation [32]. The upregulation of TMEM16A in CF glands is particularly intriguing. This phenomenon may result from the presence of an infected/inflamed environment. If so, this could mean that such conditions are more effective on glands than on the surface epithelium. Alternatively, it could be hypothesized that increased TMEM16A expression is an intrinsic response of glands to try to compensate for the absence of functional CFTR.

Summarizing, we found that bacterial components, specifically pyocyanin, stimulate TMEM16A expression and function in cultured bronchial epithelial cells. The resulting increase in Ca^2+^-activated anion secretion may be important to facilitate the release and transport of MUC5AC mucin, which is also induced by pyocyanin. TMEM16A expression is also upregulated in CF airways in vivo, particularly in submucosal glands. If this response has some beneficial effect on mucociliary clearance and other innate defense mechanisms requires further investigation. This is required to assess the importance of TMEM16A as a therapeutic target in CF and, possibly, in other lung diseases characterized by mucus hypersecretion.
